# Immune Microenvironment Change and Involvement of Circular RNAs in TIL Cells of Recurrent Nasopharyngeal Carcinoma

**DOI:** 10.3389/fcell.2021.722224

**Published:** 2021-08-06

**Authors:** Yumin Wang, Zhouying Peng, Yaxuan Wang, Yi Yang, Ruohao Fan, Kelei Gao, Hua Zhang, Zhihai Xie, Weihong Jiang

**Affiliations:** ^1^Department of Otolaryngology Head and Neck Surgery, Xiangya Hospital, Central South University, Changsha, China; ^2^Department of Nuclear Medicine, Xiangya Hospital, Central South University, Changsha, China

**Keywords:** recurrent nasopharyngeal carcinoma, TIL, immune microenvironment, circRNA, non-coding RNA

## Abstract

Nasopharyngeal carcinoma is a malignant tumor that is highly prevalent in southern China and the Southeast Asian belt. Recent studies have shown that the T cells play important regulatory roles in tumorigenesis and progression. We test TIL cell of recurrent nasopharyngeal carcinoma and primary nasopharyngeal carcinoma cell. We found that T cell change in recurrent nasopharyngeal carcinoma and primary nasopharyngeal carcinoma cell. Based on GEO database, we selected differently expressed circRNAs in nasopharyngeal carcinoma tissues. qRTPCR show that some circRNAs also highly expressed in TIL cells. In conclusion, immune microenvironment changed in recurrent nasopharyngeal carcinoma. There is involvement of circular RNAs in this progress, with should be researched further.

## Introduction

Nasopharyngeal carcinoma is a malignant tumor that is highly prevalent in southern China and Southeast Asian (Hanahan and Weinberg, 2011; Chen et al., 2019; Jiang et al., 2020). The preferred treatment for nasopharyngeal carcinoma is radiation therapy. However clinical studies have found that recurrence has become the main cause of treatment failure in nasopharyngeal carcinoma (Liu et al., 2021; Wu et al., 2021). Recent studies have shown that the process of nasopharyngeal carcinogenesis and progression is influenced by the immune microenvironment. In which T cells, as the main component of specific immunity, especially cellular immunity, play an important role in the development of tumor (Xia et al., 2021). Among the T cells, Treg, CD4 + T, CD8 + T, and other major T cell taxa play important regulatory roles in tumorigenesis and progression.

Circular RNA (circRNA) is a hot spot for cancer research in recent years. circRNA is now found to be widely involved in tumorigenesis and development (Wang et al., 2017; Zhong et al., 2018; Chen, 2020). circRNA’s modes of action include three modes of action, including ceRNA, binding to RBP proteins, and encoding small peptides. In the development of nasopharyngeal carcinoma, numerous circRNAs have been reported to be involved in a series of biological processes related to invasion and metastasis, proliferation, and resistance to radiotherapy (Zheng et al., 2016; Jin et al., 2020; Tessier et al., 2020). However, the circRNAs involved in the regulation of immune microenvironment of recurrent nasopharyngeal carcinoma, especially T-cell regulation, have not been reported yet.

In this study, we will investigate the changes in the distribution of T cells in recurrent nasopharyngeal carcinoma compared with primary nasopharyngeal carcinoma and the changes in the composition of different types of T cells. We will further investigate the expression of circRNAs in recurrent nasopharyngeal carcinoma T cells and the network of their roles in T cells. This study will be the first to reveal the alteration of T cells in nasopharyngeal carcinoma tissues and will explore the potential effect of circRNA on T cells. This study will provide new research ideas to reveal the alteration of immune microenvironment during nasopharyngeal cancer recurrence.

## Materials and Methods

### Patients and Sample

Seven nasopharyngeal carcinoma samples were obtained from patients. All samples were collected with the consent of patients and the experiments were approved by the ethics committee of Xiangya Hospital, Central South University. All fresh tissues were immersed in RNALater (Ambion, Austin, TX, United States). The tissue samples were stored in a −80°C laboratory freezer.

### Flow Cytometry to Detect T Cell

The following monoclonal antibodies and regents were purchased from BD Biosciences and added to 100 μl of cells. The panel of antibodies in were anti-CD25, anti-CD3, anti-CD4, anti-CD8, and anti-CD127. The phenotype of T reg cells were detected using anti-CD25 anti-CD127. Isotype controls with irrelevant specificities were included as negative controls. All of these cell suspensions were incubated for 20 min at room temperature. After incubating, the cells were washed and re-suspended in 200 μl of PBS. The cells were then analyzed with FACSCanto flow cytometer (BD LSRFortessaTM X-20).

### RNA Extraction and Quantitative Real-Time PCR Analyses (qRT-PCR)

Tissue RNA isolation and amplification were performed as our laboratory described previously (He et al., 2016; Mo et al., 2019a). Cell RNA was extracted using TRIzol reagent (Invitrogen, Carlbad, CA, United States). For qRT-PCR, RNA was reverse transcribed to cDNA by using a PrimeScript RT reagent Kit (Takara, Dalian, China). qRT-PCR was performed using a SYBR_Premix ExTaqII kit (Takara, Dalian, China) in the CFX96 Real-Time PCR Detection System (Bio-Rad, Hercules, CA, United States) to determine the relative expression levels of target genes. The sequences of qRT-PCR primers: circ hsa_circ_0006935 forward primer 5′-CCCTGAGTTGGTGCTGAAAAC-3′ and reverse primer 5′-AACCACGAAAGCCACCTCTG-3′; hsa_circ_0001730 forward primer 5′-CCCAATATCATCCGCCTGGA-3′ and reverse primer 5′-GCAAATTCCCTCACAGCCTCA-3′; hsa_circ_0000831 for ward primer 5′-CCTCTGGTACTTGGGGAACT-3′ and rev erse primer 5′-TCCAAAATCCCACTGTTACCAA-3′; hsa_ci rc_0031584 forward primer 5′-TGGTTACAGCTGAGAAGC CC-3′ and reverse primer 5′-TTTGTATTTCCCTCATTTCT GTGC-3′; hsa_circ_0005019 forward primer 5′-GTACAC CACCCATGAGGACG-3′ and reverse primer 5′-ACAGAT GTGTCAGAACCCTCAC-3′: GAPDH: forward primer 5’-TCACCAACTGGGACGACATG-3′ and reverse primer 5′-GTCACCGGAGTCCATCACGAT-3′; GAPDH was used as reference and normalization control.

### Public Database Profiles Download and Analysis

Nasopharyngeal carcinoma circular RNA expression data sets and the corresponding clinical data were downloaded from publicly available GEO databases. This database included 4 patients with NPC and 4 non-NPC nasopharyngeal epithelial tissues from GSE134797 (Yang et al., 2020). To find functional circRNAs in nasopharyngeal carcinoma, after downloading Nasopharyngeal carcinoma circRNA expression data from GEO database: GSE134797 (Illumina HiSeq 4000). We used Significant Analysis of Microarray (SAM) software to analyze the expression of circRNAs between the non-tumor NPE biopsies and nasopharyngeal carcinoma tissue samples in the dataset. The cut off value for differentially expressed lncRNA was set at ≥2-fold change and the false discovery ratio (FDR) was <0.05 (Mo et al., 2019a). To analyze tumor infiltration in nasopharyngeal carcinoma, after downloading Nasopharyngeal carcinoma mRNA expression data from GEO database: GSE118719 (Illumina HiSeq 4000). There are seven NPC biopsy specimens and four normal nasopharyngeal mucosal specimens in this dataset. We used Immunedeconv software to analyze the expression of circRNAs between the non-tumor NPE biopsies and nasopharyngeal carcinoma tissue samples in the dataset (Sturm et al., 2019).

## Results

### Characteristics of Nasopharyngeal Carcinoma Tissue Samples

**FIGURE 1:**
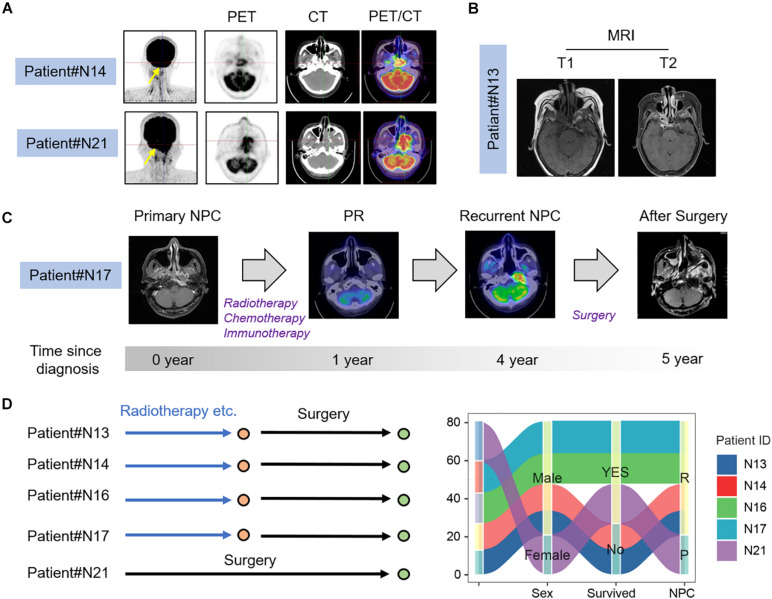
Clinical characteristics of NPC patients. **(A)** PET-CT image of recurrent and primary NPC patients; **(B)** MRI images of recurrent NPC patients; **(C)** Process of treatment for recurrent nasopharyngeal carcinoma patient. **(D)** Clinical characters and treatment history sanky map of selected NPC patients.

We selected a total of seven nasopharyngeal carcinoma tissues, including six patients with recurrent nasopharyngeal carcinoma, whose PET/CT and MRI are shown in Figures 1A,B. From PET/CT and MRI, it can be found that tumor had a recurrence after the treatment. Figure 1C shows the imaging data of #N17 patients’ illness and the whole process of treatment. The specimen and clinical data of the initial patient are shown in Figure 1D, which shows that the patient had an occupying lesion in the nasopharyngeal area. In addition, we collected surgical tissues from patients who developed osteonecrosis after radiotherapy. The PET/CT results and relevant clinical information of the patients before and after treatment can be seen in the figure. Sanky figure show treatment procedure and clinical characteristics of selected nasopharyngeal carcinoma patients (Figure 1D).

### Screening of T Cells in Nasopharyngeal Carcinoma Tissues

**FIGURE 2:**
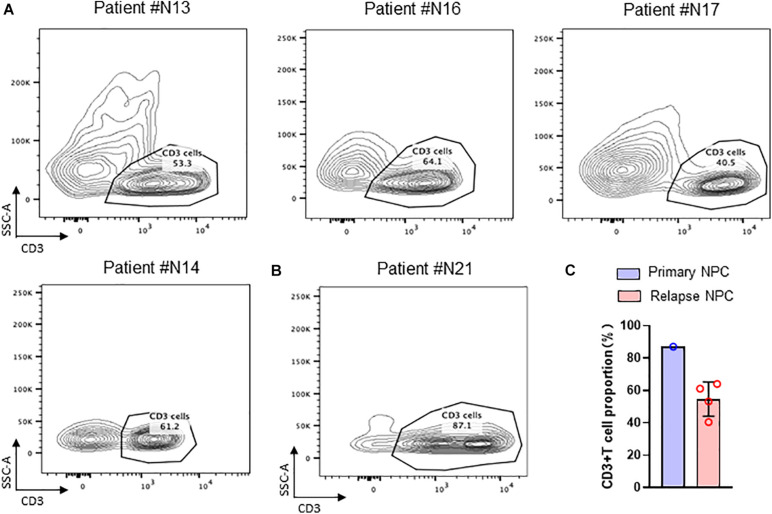
TIL cells in NPC tissue. **(A)** Flow cytometry of CD3 + T cell in recurrent NPC tissue; **(B)** Flow cytometry of CD3 + T cell in primary NPC tissue. **(C)** Statistical chart of CD3 + T cell proportion.

By flow sorting of nasopharyngeal carcinoma tissues, we screened to obtain the percentage of T cells in TIL in several nasopharyngeal carcinoma tissues. The results by using flow cytometry showed that the percentage of T cells in TIL was significantly lower in the recurrent nasopharyngeal carcinoma group compared to the initial nasopharyngeal carcinoma group by comparing the percentage of T cells in the initial nasopharyngeal carcinoma (Figure 2).

### T-Cell Distribution in Nasopharyngeal Carcinoma Tissues

**FIGURE 3:**
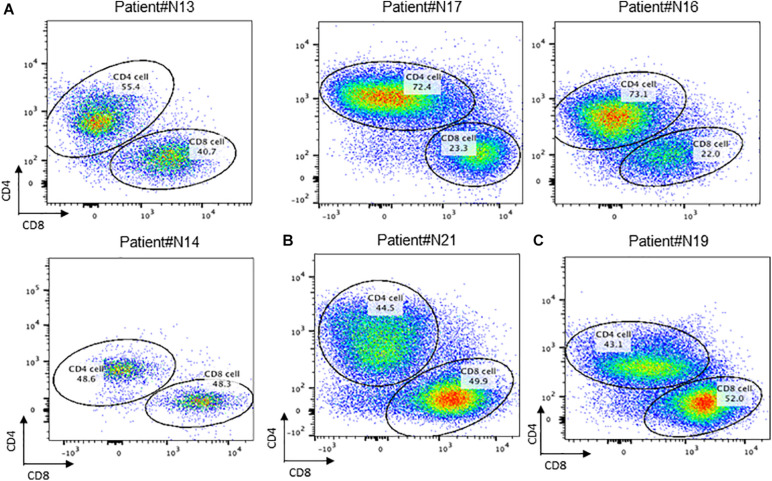
Conception of CD4+ and CD8+ cells in NPC tissue. **(A)** Flow cytometry of CD4 + T cell and CD8 + T cell in recurrent NPC tissue; **(B)** Flow cytometry of CD4 + T cell and CD8 + T cell in primary NPC tissue. **(C)** Flow cytometry of CD4 + T cell and CD8 + T cell in residual NPC tissue.

To further investigate the distribution of T cells in recurrent nasopharyngeal carcinoma tissues, we further performed flow analysis of T cell subsets in the tissues. Detection of cell surface CD molecules using flow cytometry revealed that CD4 and CD8 were used to classify the CD3-positive cell population, and the proportion of T cells accounted for by CD4 and CD8-positive cells could be obtained, respectively, and it could be found that the ratio of CD4/CD8 accounted for by CD4/CD8 was greater than 1, and some of them could even be twice as high as CD8. This is consistent with the lower value of CD4/CD8 reported in some tumor literature implying a better prognosis. Then this result, the local tumor microenvironment of recurrent nasopharyngeal carcinoma after radiotherapy is altered in the case of certain chemokines and cytokines that contribute to the accumulation of CD4 expression positive cells toward the tumor (Figure 3A).

By comparing the proportion of T cells in TILs in primary nasopharyngeal carcinoma and recurrent nasopharyngeal carcinoma, it was observed that the proportion of T cells in the recurrent nasopharyngeal carcinoma group was generally lower than that in N21, a primary nasopharyngeal carcinoma, which is consistent with the view that radiotherapy may reduce the proportion of T cells, and it has also been reported in the literature that a higher proportion of TILs in primary tumors predicts a better prognosis. However, due to the small number of patients enrolled in primary nasopharyngeal carcinoma, data are not available at this time (Figure 3B).

The ratio of CD4 and CD8 positive cells to T cells can be obtained by using CD4 and CD8 on the CD3 positive cell population circle gate. This result indicated that the local tumor microenvironment of recurrent nasopharyngeal carcinoma after radiotherapy is altered in the presence of certain chemokines and cytokines, which contribute to the accumulation of CD4 expression-positive cells toward the tumor.

**FIGURE 4:**
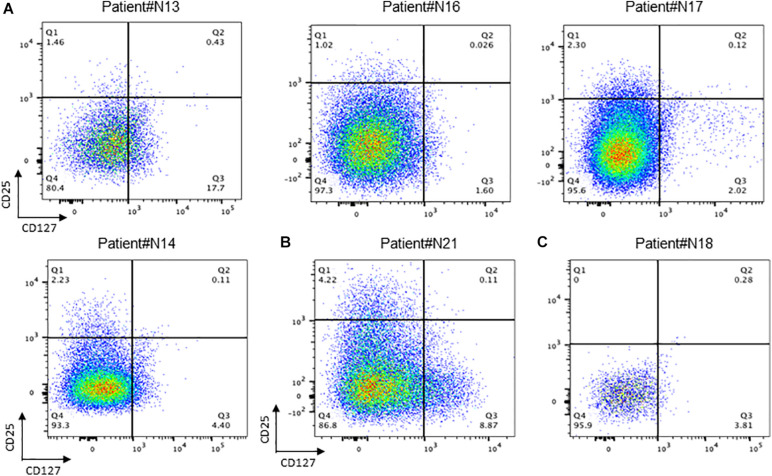
Conception of regulation T cells in NPC tissue. **(A)** Flow cytometry of CD25+/CD127+ T cell in recurrent NPC tissue; **(B)** Flow cytometry of CD25+ /CD127+ T cell in primary NPC tissue. **(C)** Flow cytometry of CD25+ /CD127+ T cell in recurrent NPC tissue with Osteonecrosis.

It can be found in Figure 4 that the percentage of T reg cells in T cells is generally lower in patients who have undergone radiotherapy than in primary nasopharyngeal carcinoma, which is contrary to what has been reported in some of the literature and needs to be investigated more thoroughly.

### Involvement of Circular RNAs in Immune Microenvironment

**FIGURE 5:**
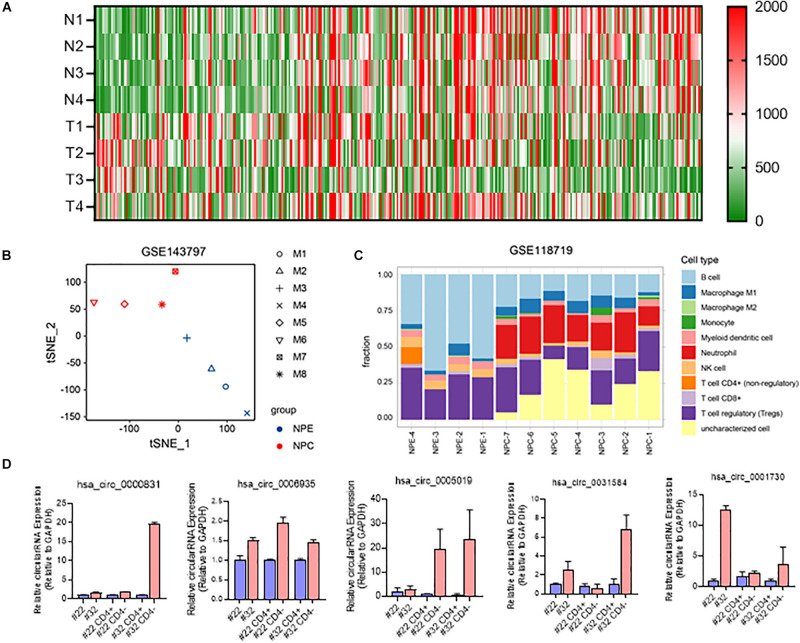
Involvement of circRNAs in NPC immune microenvironment. **(A)** Heatmap of differently expressed circRNAs in NPC and NPE; **(B)** t-SNE map of 4 NPE tissues and 4 NPC tissues based on circRNA expression; **(C)** immunocyte infiltration of 7 NPC tussues and 4 NPE tissues; **(D)** RNA expression of 5 differently expressed circRNA in NPC tissues and CD3+ cells.

As a hot topic in recent years, the expression of circRNA in T cells of nasopharyngeal carcinoma has not been reported yet. We obtained nasopharyngeal carcinoma circRNA sequencing data (GSE134797). Five differently expressed circRNAs with high expression in nasopharyngeal carcinoma tissues were obtained by differential analysis of RNAseq data (Figure 5A). CircRNA expression based tSNE map also showed that there are difference between nasopharyngeal carcinoma tissue and non-NPC nasopharyngeal epithelial tissues (Figure 5B).

Further gene immune infiltration analysis of differently expressed gene molecules, we constructed an immune infiltration cell model in both NPC and normal NPE. The results suggested that compared with primary non-NPC nasopharyngeal epithelial tissues, there is much difference in nasopharyngeal carcinoma, especially immune cell and their function (Figure 5C).

We then isolated CD4 positive and CD4 negative cells in surgically obtained samples of recurrent nasopharyngeal carcinoma. After extracting RNA from these CD4-positive and negative cells and reverse transcribing to obtain cDNA, qRTPCR was used to test the expression of selected five circRNAs in CD4-positive and negative cells obtained by screening. Results showed that these five circRNA molecules were all highly expressed in T cells (Ct value < 26) and NPC tissues (Figure 5C).

## Discussion

Nasopharyngeal carcinoma is a malignant tumor of nasopharyngeal epithelial origin, and the treatment of primary nasopharyngeal carcinoma is mainly based on radiation therapy (Wang et al., 2019; Peng et al., 2021). Although most nasopharyngeal carcinomas are initially sensitive to radiation therapy, more and more clinical applications have found that most nasopharyngeal carcinomas will develop radiation therapy resistance and recurrence. The mechanism of radiation resistance and recurrence has become a major cause of treatment failure in nasopharyngeal carcinoma. However, the mechanisms involved are still very unclear (Suarez et al., 2010; Zhang et al., 2020). Immunity is an important influencing factor in the development of nasopharyngeal carcinoma. In recent years, the concept of tumor immune microenvironment has also led to a more in-depth study of immune alterations in tumors. Tumor infiltrating cell TIL is a very popular research area in recent years, as a special cell type of T cells, there is an interaction between TIL and tumor cells.

As the main component of cellular immunity, T cells play an important function in tumor immunity. With the intensive study of immunity, T cells have been found to exist in numerous subpopulations (Li et al., 2021). As the main cell type that regulates cellular immunity, Treg plays an important function in immunity. Regulatory T cells (Treg) are a subset of T cells with significant immunosuppressive effects, characterized by a cellular phenotype expressing Foxp3, CD25, and CD4. People have found that Treg plays an important function in the development and progression of diseases such as tumors (Chen et al., 2020). It can suppress the immune response of other cells and is the primary controller of self-tolerance. Often, its absence or abnormal function leads to the development of several autoimmune-related diseases, including tumors. After immune cells, such as T cells, B cells, and NK cells, generate immune responses to recognize and remove harmful substances and thus protect the body from attack, Treg cells suppress these immune cells, including by secreting cytokines, to regulate the body’s immune homeostasis and prevent autoimmune diseases (Mo et al., 2019b; Selck and Dominguez-Villar, 2021). Our study shows that the composition of tumor T cells changes significantly during the recurrence of nasopharyngeal carcinoma, both in the number of CD4+ cells and in the CD4+/CD8+ ratio. This also suggests that T-cell subsets are significantly altered during nasopharyngeal carcinoma recurrence. As the main target cells for tumor immunotherapy, the altered composition and number of T cells suggest that the immunotherapy regimen for recurrent nasopharyngeal carcinoma may be different from that for primary nasopharyngeal carcinoma.

Circular RNA molecules play an important function in immunity. As a hot topic of non-coding RNA research in recent years, circRNAs have been found to play important functions in numerous life activities (Anczukow et al., 2015; He et al., 2016; Wang et al., 2021). The current modes of action of circRNAs include ceRNA regulation, encoding small peptides, and binding proteins to exert functions (Li et al., 2018; Wang et al., 2019; Chi et al., 2020). Although the latter two modes of action are currently uncommon, a growing number of studies suggest that circRNAs function in a variety of ways. Several studies have been reported on the function of circRNAs in T cells. Reports showing EBV coding circRNA have function in nasopharyngeal carcinoma by regulating PDL1, pathway to function. But the function of circRNA in immune cells is still very unclear. In addition circRNA has great advantages and potential as a molecular marker of tumor due to its stability brought by its special structure. Whether circRNA can be used as a marker for predicting the effect of immunotherapy is still unclear. It is a direction that deserves further exploration.

As a well-defined factor associated with nasopharyngeal carcinogenesis, EBV infection causing uncontrolled inflammation and nasopharyngeal carcinogenesis are significantly correlated. EBVs, their encoded proteins and microRNAs, and even encoded circRNAs have been shown to be important contributors to the development and progression of nasopharyngeal carcinoma. However, the involvement of circRNAs in the immune microenvironment is still very unclear. Our study, on the other hand, found that has-circ-0006935 and other circRNAs are not only highly expressed in nasopharyngeal carcinoma tissues, but also in immune cells of recurrent nasopharyngeal carcinoma tissues, even compared to CD3- cells, some circRNAs’ expression was higher in CD3+ cells. These results suggest that circRNA plays an important function in the tumor microenvironment caused by tumor recurrence, and further studies are needed to explore.

In recent years, surgery has gradually become one of the main modalities for the treatment of recurrent nasopharyngeal carcinoma (Liu et al., 2019, 2020). In turn, surgery can also have an impact on the immune status of the patient as well as the tumor microenvironment (Jiang et al., 2020; Tseng et al., 2020). Therefore the effect of surgery as a variable on the immune microenvironment of nasopharyngeal carcinoma and whether circRNA plays an important function in this process. This is a question that deserves to be explored in depth.

In conclusion, we found that the ratio of T cells in the tumor microenvironment may be altered in recurrent nasopharyngeal carcinoma compared to primary nasopharyngeal carcinoma. Alterations in Treg and CD4+/CD8+ ratios may also be found. circRNA is highly expressed in tumor-associated T cells and may play an important function.

## Data Availability Statement

The raw data supporting the conclusions of this article will be made available by the authors, without undue reservation.

## Ethics Statement

The studies involving human participants were reviewed and approved by Xiangya Hospital, Central South University. The patients/participants provided their written informed consent to participate in this study. Written informed consent was obtained from the individual(s) for the publication of any potentially identifiable images or data included in this article.

## Author Contributions

YuW and ZP contributed to all of the literature review in this work. YaW, YY, and RF collected and analyzed the data of this article. WJ participated in the process of finalizing the final manuscript. All authors have read and approved the manuscript.

## Conflict of Interest

The authors declare that the research was conducted in the absence of any commercial or financial relationships that could be construed as a potential conflict of interest. The handling editor declared a shared affiliation, though no other collaboration, with the authors at the time of the review.

## Publisher’s Note

All claims expressed in this article are solely those of the authors and do not necessarily represent those of their affiliated organizations, or those of the publisher, the editors and the reviewers. Any product that may be evaluated in this article, or claim that may be made by its manufacturer, is not guaranteed or endorsed by the publisher.
